# Perception and Attitudes Toward PPE-Related Waste Disposal Amid COVID-19 in Bangladesh: An Exploratory Study

**DOI:** 10.3389/fpubh.2020.592345

**Published:** 2020-11-13

**Authors:** S. M. Didar-Ul Islam, Mariam Binte Safiq, Md. Bodrud-Doza, Mohammed A. Mamun

**Affiliations:** ^1^Department of Environmental Sciences, Jahangirnagar University, Dhaka, Bangladesh; ^2^School of Environment, Tsinghua University, Beijing, China; ^3^Centre for Health Innovation, Networking, Training, Action and Research - Bangladesh, Dhaka, Bangladesh; ^4^Saphena Women's Dental College and General Hospital, Dhaka, Bangladesh; ^5^Climate Change Programme (CCP), BRAC, Dhaka, Bangladesh; ^6^Department of Public Health and Informatics, Jahangirnagar University, Dhaka, Bangladesh

**Keywords:** COVID-19 pandemic, PPE waste disposal, environmental pollution, environmental health risk, public attitudes and practices, medical waste in Bangladesh

## Abstract

Personal protective equipment (PPE) is an essential item to protect from exposure to infectious pathogens or contaminants, which is frequently used at health care settings and public spheres since the coronavirus disease 2019 (COVID-19) outbreak. There is no prior study investigating public perception and attitudes toward PPE-related waste disposal in Bangladesh. Hence, an online survey was carried out among 1,303 Bangladeshi adult residents to explore the issue. Results stated that face mask and hand gloves were the widely used PPE, where around 45.50% mask and 31.60% hand gloves were disposable. Approximately 94.50% of the participants percepted to use at least one type of PPE while going outside. Only 18.65% of the respondents percepted to burn the PPE-related waste, while most of them reported other less protective disposal measures. Females, urban residents, and participants with higher education were found to have better perception and attitudes toward PPE-related waste disposal. To the best of the authors' knowledge, being the first exploratory study in the country, the present findings are anticipated to be helpful at policy levels with respect to arranging awareness programs.

## Introduction

Since the coronavirus disease 2019 (COVID-19) outbreak, personal protective equipment (PPE) (e.g., face masks, gloves, goggles, gowns, etc.) is being widely used in health care settings and public spheres, which rapidly accumulates potential infectious waste in the solid waste streams throughout the world (1). Proper disposal of these wastes is essential for the control of the reemergence of viral infection, and environmental protection (2), as well as to meet the Sustainable Development Goals, especially SDG3, SDG6, SDG8, SDG12, and SDG13 (3). Bangladesh reported the first COVID-19 case on March 8, 2020, and as of September 7, 2020, a total of 327,359 cases are reported (4). To control the COVID-19 transmission, the government encouraged people to use PPE through public awareness programs, and a rule concerning mandatory mask use was enforced on July 21, 2020 (5). The country is already alleged to have the worst waste management system, and the sudden rise of COVID-19-related waste load and its improper disposal increased the risk of re-transmission and had consequences on the environment as well (1, 6, 7).

**Figure 1:**
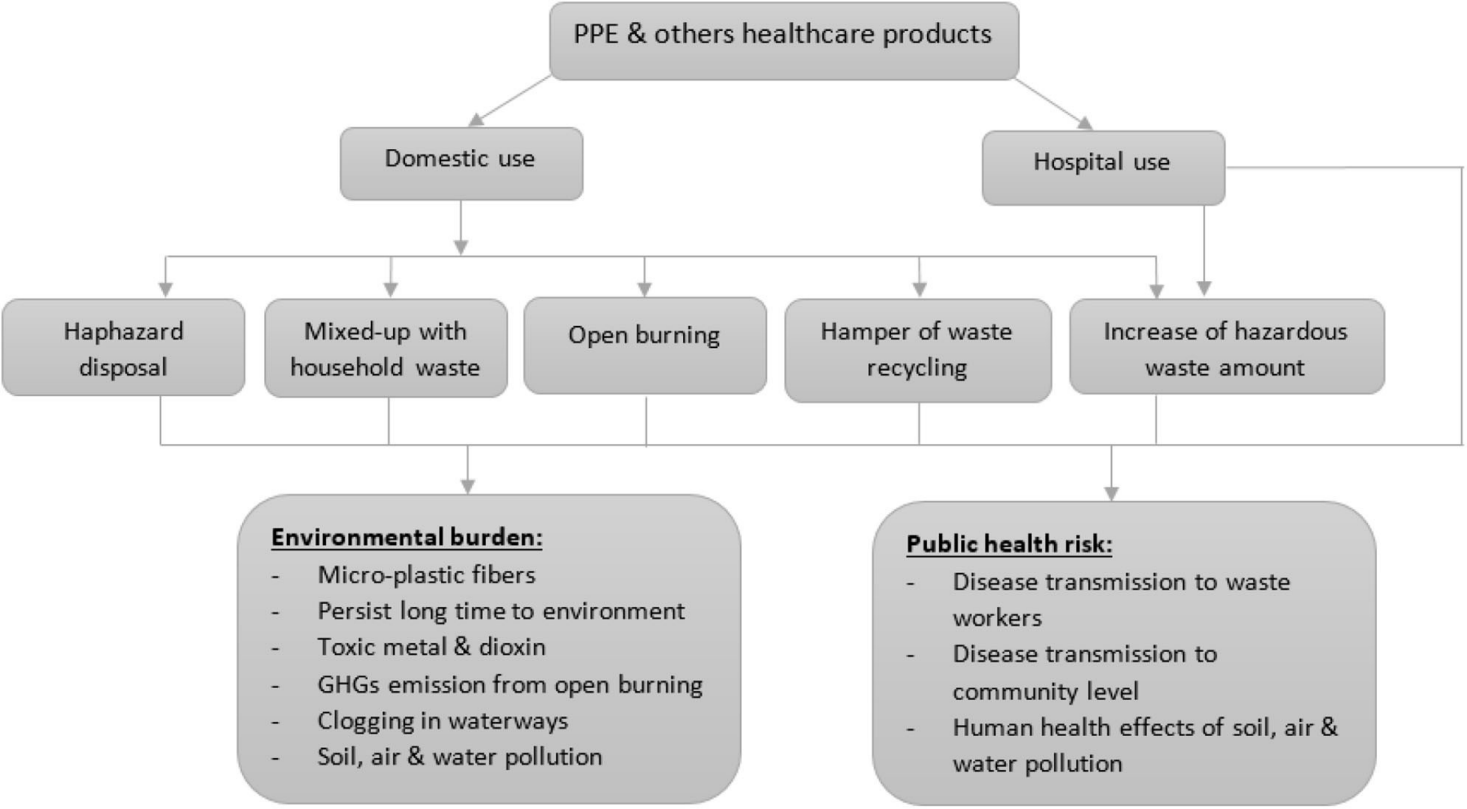
Figure showing the environmental and public health risk of improper disposal of personal protective equipment (PPE) and others healthcare wastes (2, 11).

For having no recognized treatment available for COVID-19 patients, along with other public health measures, PPE is being recommended for use to escape from the potential virus infection (1, 8). Consistent with personal safety measures, Bangladeshi people have not fled out of the scenario. But, proper disposal of PPE-related waste is indispensable to reduce disease transmission (1, 6, 9). The haphazard disposal of these wastes may create clogging in waterways (e.g., municipal drain, canal, etc.) and enhance environmental pollution load, especially in poor urban areas (10). Plastic-based face masks and other PPE are known as a potential source of micro-plastic fibers in the environment (11). It is suggested that proper disposal and segregation of household waste with plastic-based healthcare waste and mix-up of these wastes increase the risk of disease transmission to waste workers (2). Figure 1 illustrates the probable environmental and human health risk of PPE-related waste. Therefore, it is urgent to properly dispose of used PPE to lessen unwanted infectious sources (2, 6). Therefore, the present study, for the first time in Bangladesh, investigated the perception and attitudes toward PPE-related waste disposal, which may help government authorities to rethink policy levels.

## Methods

An online survey was conducted from May 20 to June 19, 2020, among a total of 1,303 Bangladeshi adult residents. Consistent with the study aims, a self-administered questionnaire was applied based on the national and international guidelines and literature regarding PPE waste disposal. The questionnaire included sociodemographics and safety equipment's use and disposal perception- and attitude-related questions. PPE waste disposal perception and attitudes were assessed with a total of five items based on a 5-point Likert scale (1 = strongly disagree to 5 = strongly agree), whereas overall score was based on summing all items (what the extract items were asked is presented in the Table 1 footnote). The data were analyzed through Microsoft Excel (2010) and Statistical Package for the Social Sciences (version 25.0). Descriptive statistics such as frequency and percentage were used along with the ANOVA tests to test for PPE use perception and attitude mean differences with the variables. The level of statistical significance was *p* < 0.05 for all tests. Frequency and descriptive statistics were used to determine mean scores and standard deviations of the study variables.

**Table 1:** Mean difference of personal protective equipment (PPE)-related waste disposal perception attitudes.

**Variables (*n*; %)**	**Overall perception and attitudes**	**P&A-1**	**P&A-2**	**P&A-3**	**P&A-4**	**P&A-5**
Total mean ± SD	17.754 ± 3.342	4.673 ± 0.749	3.399 ± 1.357	3.291 ± 1.459	2.360 ± 1.275	4.031 ± 1.161
**Gender**						
Male (745; 57.20%)	17.431 ± 3.475***	4.663 ± 0.766	3.258 ± 1.351***	3.268 ± 1.456	2.323 ± 1.296	3.918 ± 1.240***
Female (558; 42.80%)	18.186 ± 3.106	4.686 ± 0.727	3.589 ± 1.342	3.321 ± 1.463	2.408 ± 1.247	4.181 ± 1.029
**Age group**						
18–30 years (1,138; 87.3%)	17.728 ± 3.374	4.676 ± 0.751**	3.395 ± 1.363	3.241 ± 1.465***	2.401 ± 1.286*	4.016 ± 1.159
31–40 years (99; 7.6%)	18.37 ± 3.151	4.778 ± 0.581	3.586 ± 1.317	3.747 ± 1.296	2.111 ± 1.186	4.151 ± 1.248
41–50 years (31; 2.4%)	17.645 ± 3.136	4.677 ± 0.599	3.419 ± 1.385	3.581 ± 1.500	2.000 ± 1.211	3.967 ± 1.277
More than 51 years (35; 2.7%)	16.943 ± 2.786	4.286 ± 1.073	3.029 ± 1.175	3.371 ± 1.457	2.057 ± 1.109	4.200 ± 0.867
**Residence**						
Rural (365; 28.0%)	16.644 ± 3.573***	4.430 ± 0.957***	3.096 ± 1.300***	3.008 ± 1.409***	2.564 ± 1.204***	3.545 ± 1.256***
Urban (938; 72.0%)	18.187 ± 3.145	4.768 ± 0.626	3.518 ± 1.360	3.401 ± 1.464	2.280 ± 1.294	4.219 ± 1.065
**Education**						
Primary (23; 1.8%)	15.913 ± 2.678*	4.174 ± 1.072*	2.869 ± 1.140**	2.826 ± 1.614	2.174 ± 1.193	3.869 ± 0.967
Secondary (50; 3.8%)	17.420 ± 3.643	4.640 ± 0.875	3.240 ± 1.302	3.120 ± 1.466	2.500 ± 1.344	3.920 ± 1.047
Higher secondary (318; 24.4%)	17.616 ± 3.194	4.679 ± 0.722	3.286 ± 1.354	3.211 ± 1.442	2.384 ± 1.292	4.056 ± 1.116
Graduate (650; 49.9%)	17.917 ± 3.225	4.692 ± 0.720	3.521 ± 1.343	3.295 ± 1.461	2.377 ± 1.265	4.031 ± 1.156
Postgraduate (262; 20.1%)	17.744 ± 3.735	4.668 ± 0.783	3.313 ± 1.398	3.450 ± 1.450	2.279 ± 1.278	4.034 ± 1.266
**Occupation**						
Business (30; 2.3%)	17.700 ± 4.036	4.700 ± 0.794***	3.200 ± 1.186	3.533 ± 1.408	2.400 ± 1.003	3.867 ± 1.224
Service (219; 16.8%)	17.612 ± 3.428	4.667 ± 0.780	3.365 ± 1.389	3.425 ± 1.458	2.210 ± 1.246	3.945 ± 1.312
Student (832; 63.9%)	17.829 ± 3.301	4.686 ± 0.733	3.454 ± 1.354	3.215 ± 1.479	2.433 ± 1.304	4.041 ± 1.127
Teacher (42; 3.2%)	18.381 ± 3.882	4.881 ± 0.328	3.452 ± 1.451	3.643 ± 1.574	2.238 ± 1.411	4.167 ± 1.286
Housewife (48; 3.7%)	17.312 ± 3.149	4.187 ± 1.065	3.083 ± 1.235	3.500 ± 1.288	2.375 ± 1.248	4.167 ± 0.975
Unemployed (73; 5.6%)	17.479 ± 3.420	4.671 ± 0.765	3.219 ± 1.315	3.411 ± 1.245	2.274 ± 1.133	3.904 ± 1.227
Others (59; 4.5%)	17.508 ± 2.873	4.746 ± 0.575	3.305 ± 1.417	3.169 ± 1.440	2.051 ± 1.121	3.237 ± 0.953

***p < 0.001; **p < 0.01; *p < 0.05.

*P&A-1, wearing PPE (e.g., mask, hand gloves, etc.) while going outside of home; P&A-2, disposing PPE and other healthcare waste in separate covered bins or bags; P&A-3, disposing PPE and other healthcare waste in household waste bins; P&A-4, burning PPE and other healthcare waste individually; P&A-5, disposing PPE and other healthcare waste in community containers or disposal areas*.

## Results

A total of 1,303 responses were recorded in this study (mean age = 27.16 ± 7.78 years); 57.20% were male (*n* = 745), and 72% were from urban or semi-urban areas (*n* = 937). Most of the respondents were educated, and education levels were graduate level or above (70.0%), and 24.40% had higher secondary education level. Nearly 64% of them were students, along with other professions including teacher (3.20%), service holder (16.80%), businessman (2.30%), housewife (3.70%), unemployed (5.60%), and others (4.50%) (Table 1).

**Figure 2:**
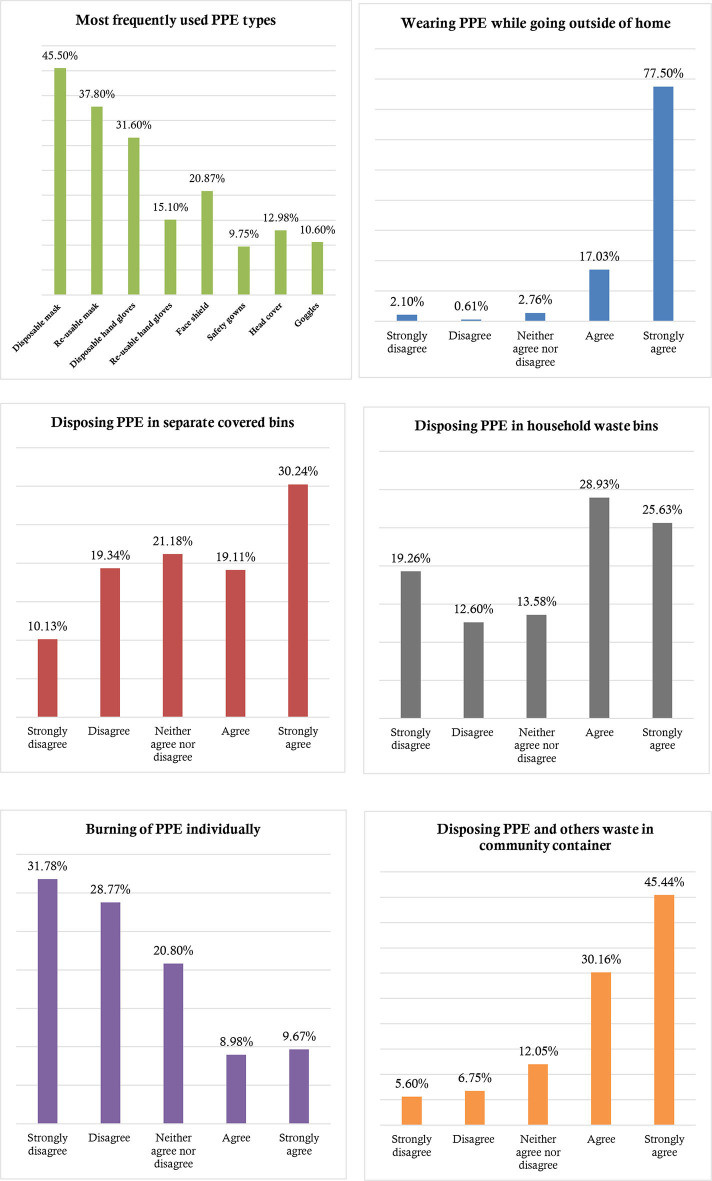
Distribution of personal protective equipment (PPE) use and PPE-related waste disposal perception and attitudes.

Among the available PPE, face mask and hand gloves were highly used. Of these, nearly 45.50 and 31.60% of people used disposable face masks and hand gloves, respectively. Approximately 94.50% of the participants percepted to use at least any type of protective equipment as a preventive measure of COVID-19 while going outside of home for working, shopping, or any other purpose. Only half of the respondents (49.35%) perceived to disposed of the used mask, hand gloves, and others healthcare waste in separate covered bins or bags, whereas 54.56 and 75.60% reported to have the attitudes of disposing PPE in household bins and in the community container or disposal area, respectively. Only 18.65% of participants percepted to burn their used mask, hand gloves, tissues, and other bio-waste to reduce disease transmission (Figure 2).

In Table 1, the relationship between PPE-related waste disposal perception and attitudes and sociodemographics are presented. Within the total sample, the PPE-related waste disposal perception and attitude mean score was 17.754 (± 3.342). However, PPE waste disposal perception and attitude mean scores were higher in female gender (18.186 ± 3.106 vs. 17.431 ± 3.475; *f* = 16.502, *p* < 0.001), urban residence (18.187 ± 3.145 vs. 16.644 ± 3.573; *f* = 58.473, *p* < 0.001), and higher education level (*f* = 2.402, *p* = 0.048). Besides, significant mean differences of perception and attitude components, i.e., disposing in separate covered bins or bags (*f* = 19.360, *p* < 0.001) and disposing in community containers or disposal areas (*f* = 16.535, *p* < 0.001) were also found to be higher in females. Whereas, participants from urban areas highly percepted across all of the components. Lastly, education level was significantly associated with wearing PPE while going outside (*f* = 2.707, *p* = 0.029) and disposing PPE-related waste in covered bins (*f* = 3.207, *p* = 0.012), whereas it was only wearing PPE while going outside that associated with occupation status (*f* = 4.103, *p* < 0.001; Table 1).

## Discussion

To the best of the authors' knowledge, the present study for the first time provides an initial observation on PPE-related waste disposal perception and attitudes amid the COVID-19 outbreak among the Bangladeshi sample. Based on the findings, a higher portion of the participants reported to have the perception and attitudes of disposing PPE-related waste within household waste and in community containers or disposal areas, which may be negligibly effective against virus reinfection for the country. Bangladesh has been reported to mismanage handling healthcare waste in either household or community areas despite proper rules and regulations (6). As a result, healthcare waste is mostly disposed of in unauthorized places without any separation or proper treatment by untrained, unprotected, and unaware cleaners (6).

Higher literacy is commonly regarded as the protective factor against occurring negative effects; similar assertions can be made for COVID-19-related issues. For instance, the study reported that higher education, more specifically, literacy related to COVID-19, increases the positive attitudes and practices toward the COVID-19 issue that are reported in other countries like the present finding (12, 13). Besides, the urban residents are reported to have more positive PPE-related waste disposal perception and attitudes, which can be explained by the sociodemographic condition of the country. That is, in Bangladesh, the urban people's literacy rate is far higher than the rural ones as reported by UNESCO (14). An Egyptian study observed the positive effect of safety and waste management literacy on the laboratory technician's knowledge, attitudes, and practices after implementing an intervention program (15), which reflects the urgent need of literacy awareness programs in Bangladeshi people. Moreover, it is found that females are more concerned regarding disposing of PPE-related waste compared to males. This finding may be because of their responsibilities of taking care of the family members' PPE-related waste disposal. Besides, females are usually considered as more cautious than males in terms of infectious disease prevention practices, e.g., hand hygiene, PPE use, etc., that is reported in other countries for their higher positive attitudes toward COVID-19 issues (12, 16).

Since the COVID-19 outbreak, biomedical waste generation rate has increased globally, including Bangladesh, which creates extra public health burden and becomes a challenge to waste management authorities (17). It is reported that, on average, 1.63–1.99 kg/bed/day medical waste is generated in Dhaka City, the capital of Bangladesh, whereas there are nearly 141,903 hospital beds in the country (6). Approximately, 40,000 informal waste collectors work across the country; they are at high risk of COVID-19 infection due to lack of adequate protection (6, 7, 18). Poor management of COVID-19 wastes in Bangladesh increases the risk of infection and environmental hazards. Polypropylene is the common material of protective equipment like N-95 masks, and Tyvek is used for protective suits, hand gloves, and medical face shields, which can persist for a long time and pollute the environment (1). Due to the disruption of routine municipal waste management and plastic waste recovery and recycling activities for the pandemic, it increases the landfilling and environmental pollutants like dioxins and toxic metals (1, 11).

As aforementioned, improper PPE-related waste disposal and management can be the source of reemergence of the virus infection. Therefore, ensuring public better attitudes and practices toward PPE-related waste disposal along with the time-oriented policy should be implemented. The present findings, being an initial observation, may help policy makers in facilitating public awareness programs.

## Data Availability Statement

The raw data supporting the conclusions of this article will be made available by the authors, without undue reservation.

## Ethics Statement

The studies involving human participants were reviewed and approved by Institute of Allergy and Clinical Immunology of Bangladesh, Dhaka, Bangladesh. The patients/participants provided their online informed consent to participate in this study.

## Author Contributions

SI, MB-D, and MM conceptualized the study, implemented the project, collected data, and wrote the first draft. MS and MM reanalyzed the data, interpreted the data, rewrote the draft, and addressed the reviewer comments. All authors approved the final version.

## Conflict of Interest

The authors declare that the research was conducted in the absence of any commercial or financial relationships that could be construed as a potential conflict of interest.
